# Regulation of *MYCN *expression in human neuroblastoma cells

**DOI:** 10.1186/1471-2407-9-239

**Published:** 2009-07-18

**Authors:** Joannes FM Jacobs, Hans van Bokhoven, Frank N van Leeuwen, Christina A Hulsbergen-van de Kaa, I Jolanda M de Vries, Gosse J Adema, Peter M Hoogerbrugge, Arjan PM de Brouwer

**Affiliations:** 1 Department of Tumor Immunology, Nijmegen Centre for Molecular Life Sciences, Nijmegen, the Netherlands; 2 Department of Pediatric Hemato-Oncology, Radboud University Nijmegen Medical Centre, Nijmegen, the Netherlands; 3 Department of Human Genetics, Radboud University Nijmegen Medical Centre, Nijmegen Centre for Molecular Life Sciences, Donders Institute for Brain, Cognition and Behaviour, P.O. Box 9101, 6500 HB Nijmegen, the Netherlands; 4 Department of Bloodtransfusion and Transplantation Immunology, University Nijmegen Medical Centre, Nijmegen, the Netherlands; 5 Department of Pathology, Radboud University Nijmegen Medical Centre, Nijmegen, the Netherlands

## Abstract

**Background:**

Amplification of the *MYCN *gene in neuroblastoma (NB) is associated with a poor prognosis. However, *MYCN*-amplification does not automatically result in higher expression of *MYCN *in children with NB. We hypothesized that the discrepancy between *MYCN *gene expression and prognosis in these children might be explained by the expression of either MYCN-opposite strand (*MYCNOS*) or the shortened MYCN-isoform (*ΔMYCN*) that was recently identified in fetal tissues. Both *MYCNOS *and ΔMYCN are potential inhibitors of MYCN either at the mRNA or at the protein level.

**Methods:**

Expression of *MYCN, MYCNOS *and *ΔMYCN *was measured in human NB tissues of different stages. Transcript levels were quantified using a real-time reverse transcriptase polymerase chain reaction assay (QPCR). In addition, relative expression of these three transcripts was compared to the number of *MYCN *copies, which was determined by genomic real-time PCR (gQPCR).

**Results:**

Both *ΔMYCN *and *MYCNOS *are expressed in all NBs examined. In NBs with *MYCN*-amplification, these transcripts are significantly higher expressed. The ratio of *MYCN:ΔMYCN *expression was identical in all tested NBs. This indicates that *ΔMYCN *and *MYCN *are co-regulated, which suggests that ΔMYCN is not a regulator of MYCN in NB. However, the ratio of *MYCNOS:MYCN *expression is directly correlated with NB disease stage (*p *= 0.007). In the more advanced NB stages and NBs with *MYCN*-amplification, relatively more *MYCNOS *is present as compared to *MYCN*. Expression of the antisense gene *MYCNOS *might be relevant to the progression of NB, potentially by directly inhibiting *MYCN *transcription by transcriptional interference at the DNA level.

**Conclusion:**

The *MYCNOS:MYCN*-ratio in NBs is significantly correlated with both *MYCN*-amplification and NB-stage. Our data indicate that in NB, MYCN expression levels might be influenced by *MYCNOS *but not by ΔMYCN.

## Background

Neuroblastoma cells (NBs) that carry an amplified *MYCN *gene are extremely malignant. However, *MYCN-*amplification does not automatically result in higher expression of *MYCN *in children with NB [1-3]. Thus, it has been suggested that the aggressive phenotype of *MYCN *amplified NBs may be explained by higher expression levels of other genes co-amplified with *MYCN*, since the amplified unit of DNA can be up to 1 Mb. To date, three genes have been identified that are frequently co-amplified with *MYCN *in NBs: *DDX1 *in 50% of the cases, *NAG *in 20% of the tumours, and *MYCNOS *in all cases [4,5]. All three genes demonstrate increased transcript expression when co-amplified in NB cell lines, indicating that they may contribute to tumour phenotype. However, survival analyses in a large study using 75 *MYCN*-amplified tumours indicate that neither amplified *DDX1 *nor *NAG *have an additional adverse effect on the prognosis of the patients [6].

Natural antisense transcripts are abundant in eukaryotic genomes [7-9]. In human, more than 1600 natural antisense transcript are predicted to be present [10]. They can influence gene expression on the DNA level by transcriptional interference, on the transcript level by RNA interference and RNA editing, or direct splicing by RNA masking [11,12]. *MYCNOS *is the antisense transcript of *MYCN *[13] and shows overlap with the first exon of *MYCN*. This antisense transcript could be involved in modulating the expression of *MYCN *by any of the mechanisms mentioned above. Antisense transcripts are considered to be relevant to the development and progression of tumours [14-16], but until now, only antisense *HIF-1α *RNA has been shown to be a marker for prognosis in human breast cancer [17].

Recently, we reported a fetal MYCN splice variant (*ΔMYCN*) lacking exon 2 [18]. The *ΔMYCN *transcript is expressed in several fetal tissues and contains the acidic region, nuclear localization signal, the basic helix-loop-helix and leucine-zipper domains but lacks the transactivation domain. It has been suggested that the ΔMYCN protein may serve as an obligate dimerization partner for MYCN to convey transcriptional activation or repression.

In this report we analysed whether expression of *ΔMYCN *and *MYCNOS *influence *MYCN *expression levels in NBs of different disease stages.

## Methods

### Tumour material

Sixteen fresh-frozen NBs were obtained from at the Department of Pathology at the Radboud University Nijmegen Medical Centre. All NBs were derived from pediatric patients (0 to 6-years-old) diagnosed at the Department of Pediatric Hemato-Oncology. Sections of the frozen samples were stained with hematoxylin-eosin and reviewed by the pathologist to verify tumour histology and to evaluate the percentage of tumour cells. Samples were only considered for study if the contents of tumour cells was ≥ 75%. Six out of 16 NBs had *MYCN*-amplification as shown by southern blot and/or fluorescent in situ hybridization (FISH). All samples were anonymized prior to this study, and the research program was approved by the local ethics committee (Commissie Mensgebonden Onderzoek Regio Arnhem-Nijmegen).

### DNA isolation, RNA isolation and cDNA synthesis

Tumour samples were aliquoted in two parts to isolate both DNA and RNA. Total DNA was isolated with the QIAamp isolation-kit (Qiagen, Hilden, Germany) according to the manufacturer's protocol. All samples were RNAse treated. Total RNA was isolated with TriZol reagent (Invitrogen, Carlsbad, CA, USA) and treated with Deoxyribonuclease I (Dnase I; Invitrogen). DNase-treated RNA was reverse-transcribed using oligo(dT) primers with the SuperScript First-Strand Synthesis System (Invitrogen).

### PCR

**Figure 1:**
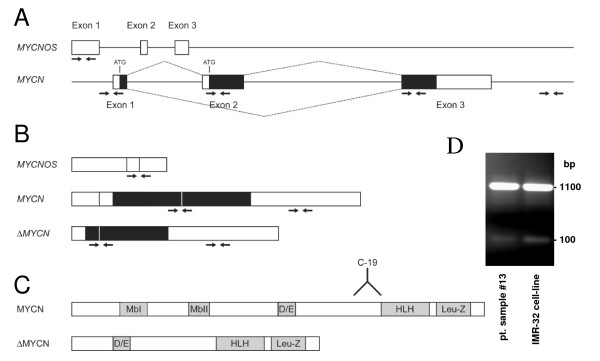
**Schematic overview of (A) genomic organization, (B) transcripts, and (C) protein isoforms of *MYCN *and *MYCNOS***. The localization of primer sites (small arrows) and the C-19 antibody epitope are indicated. (D) RT-PCR with primers on exon 1 and 3 on NB cDNA of patient 13 and NB cell line IMR-32 give products of 1007 bp (*MYCN*) and 100 bp (*ΔMYCN*). The identity of both products was verified by sequence analysis.

*MYCN *and splice variants were amplified from cDNA by using the GC-RICH PCR System (Roche Applied Science, Almere, The Netherlands). Primers were developed by the primer3 program (http://frodo.wi.mit.edu/cgi-bin/primer3/primer3_www.cgi[19]). Primer sequences are shown in table 1 and the position of the primers in *MYCN *are depicted in figure 1. PCR products were sequenced using the ABI PRISM BigDye Terminator Cycle Sequencing V2.0 Ready Reaction Kit and analysed with the ABI PRISM 3730 DNA analyser (Applied Biosystems, Foster City, CA, USA).

**Table 1:** Primer-sequences of primers used in this report.

Gene of interest	Genbank ID	Forward primer	Reverse primer
**QPCR**			

*MYCN*	NM_005378.4	5'-cacaaggccctcagtacctc-3'	5'-accacgtcgatttcttcctc-3'
*ΔMYCN*	Not present	5'-cagaatcgcctccggatc-3'	5'-cgcttctccacagtgacca-3'
MYCNOS	S49953.1	5'-tccgacagctcaaacacagac-3'	5'-ccagctttgcagccttctc-3'
*MYCN *total	NM_005378.4	5'-cataaggggtttgccatttg-3'	5'-ctaatactggccgcaaaagc-3'
*GUSB*	NM_000181.1	5'-agagtggtgctgaggattgg-3'	5'-ccctcatgctctagcgtgtc-3'
*TFRC*	NM_003234.1	5'-gttcttctgtgtggcagttcag-3'	5'-caggctgaaccgggtatatg-3'
*RNFIII*	NM_017610.6	5'-gcagaatgcagcagaagttg-3'	5'-ccattcttgcagaagtggttg-3'
			
**gQPCR**			

*MYCN *(exon 1)	NM_005378.4	5'-ccgggtgtgtcagatttttc-3'	5'-tccaacacagttcccaggag-3'
*MYCN *(exon 2)	NM_005378.4	5'-gatctgcaagaacccagacc-3'	5'-ccgccgaagtagaagtcatc-3'
*MYCN *(exon 3)	NM_005378.4	5'-gttcctcctccaacaccaag-3'	5'-aggcatcgtttgaggatcag-3'
*MYCN *(3'-UTR)	NM_005378.4	5'-taccaggtgcaggagagacc-3'	5'-agcccaagtagccaagacac-3'
*MYCNOS*	S49953.1	5'-aagaagggtagtccgaaggtg-3'	5'-gaaactggaaacatccagagg-3'
*CFTR*	NM_000492.3	5'-gggtcttgataaatggcttcc-3'	5'-tctggcttgcaaaacacaag-3'
*TBX22*	NM_016954.2	5'-tttaccggctcctgaaagac-3'	5'-aaccgctttttgaattggtg-3'
*SLC16A2*	NM_006517.2	5'-cttcttcgtccctctgatgc-3'	5'-tcaggggccaacatcttatc-3'
			
		*Cloning*	

*MYCNOS*	S49953.1	5'-agggggtggtggcgaggc-3'	5'-gtagctcgcacttatttatttat-3'

### QPCR and gQPCR

QPCR and gQPCR was performed by SYBR Green-based quantification (Bio-Rad, Veenendaal, The Netherlands). PCRs were performed on an iCycler (MyiQ single color Real-Time detection System, Bio-Rad, Veenendaal, The Netherlands). Sequences of the primers used to quantify cDNA transcript levels and genomic DNA are shown in table 1 and the position of the primers is depicted in figure 1. PCR products were between 80- and 100-bp. Validation of the primer pairs and (g)QPCR experiments were performed as described previously [20,21]. Differences in expression of a gene of interest or in genomic DNA copy number between two samples were calculated by the comparative Ct or 2^ΔΔCt ^method [22,23]. Hoebeeck et al. described and validated a similar assay for the determination of MYCN copy numbers in tumor samples [24].

### Antibody coupling and immunoprecipitation

NB-samples were homogenized in RIPA-buffer (50 mM Tris-HCl pH 7.4, 0.2% sodium dodecyl sulfate (SDS), 0.2% sodium deoxycholate, 1% triton X-100, 1 mM EDTA) supplemented with 1 mM DTT, 1 mM PMSF, aprotinin 2 μg/ml and leupeptin 2 μg/ml. Total protein concentration was determined according to the Bradford method (Bio-Rad Laboratories, Hercules, CA, USA).

For immunoprecipitation, 5 μg C-19 mAb (Santa Cruz Biotechnology, Heidelberg, Germany) was coupled to Prot A sepharose CL-4B beads (Pharmacia Biotechnologies, Uppsala, Sweden) for 1 hour at 4°C to). NB-lysates were precleared O/N with 50 μl packed Prot A sepharose CL-4B beads. To the precleared lysates, 20 μl C-19-coupled beads was added and incubated for 24 h at 4°C. Subsequently, the beads were washed with PBS and resuspended in SDS sample buffer and stored at -80°C until SDS-polyacrylamide gel electrophoresis (PAGE).

### SDS-PAGE and Western blotting

Samples (30 μg homogenized NB-sample or 20 μl precipitation beads) were separated a 10% polyacrylamide gels and transferred to nitrocellulose (Hi-bond, Amersham Biosciences, Little Chalfont, UK). Ponceau S staining was used to confirm that equal amounts of protein were loaded in each lane (additional file 1). The nitrocellulose blot was blocked with 1% BSA in 20 mM Tris-HCl pH 7.4 and 0.1% Tween (Tris-buffered saline/Tween 20; TBS-T) for 1 h. The blot was washed for 5 min with TBS-T followed by incubation with the C-19 anti-MYCN antibody 1:200 diluted in TBS-T for 1 h at RT. After washing 3× with TBS-T, the blot was incubated with HRP-conjugated swine-anti-rabbit antibody (1:5000 diluted in TBS-T) for 1 h at RT. Subsequently, the blot was washed and incubated for 1 min with ECL substrate (Amersham Biosciences, Little Chalfont, UK) and exposed to film (Kodak, Rochester, NY, USA).

### Overexpression of MYCNOS in the NB cell line IMR-32

Primers for the amplification of *MYCNOS *were developed by using the primer3 program (Table 1). *MYCNOS *was amplified from DNA isolated from a healthy control using the GC RICH PCR System (Roche, Woerden, The Netherlands). Subsequently, *MYCNOS *was cloned into the Gateway donor vector pDONR-201 (Invitrogen). Using the Gateway cloning system, *MYCNOS *was subsequently subcloned into the pcDNA3 expression vector and integrity of the construct was validated by sequence analysis. IMR-32 NB cells at 50% confluence in a 25 cm^2 ^flask were co-transfected with 8 μg pcDNA3-*MYCNOS *and C1-*GFP *using 120 μl lipofectamine (Invitrogen) in 3 ml Opti-MEM-I for 20 min at RT.

### Statistical analysis

Statistically significant differences in expression of *MYCN*-transcripts between NBs with or without *MYCN*-amplification were calculated with Students' T-test. Correlation of *MYCN*-transcipt expression with disease stage was calculated using the Spearman rank correlation and correlation of MYCN-transcripts with the *MYCN*-amplification numbers was calculated with the Pearson correlation test. All statistical tests were two-sided, significance was determined as p < 0.05.

## Results

### Patient characteristics

We analysed fresh-frozen NBs from 16 pediatric patients (age range: 0–6 years old). The NBs are classified according to the Children's Cancer Group Neuroblastoma Staging System [25] and treated according to Pediatric Oncology Group-protocols (Table 2). Six out of 16 NBs carried a *MYCN*-amplification initially detected by Southern blot and/or FISH.

**Table 2:** Patient characteristics.

Pt	**Age**^a^	Sex	Diagnosis	Localization	**Histology**^b^	**MYCN**^c^	Treatment and follow-up	Status
*1*.	3 mnts	F	NB IV S	Adnex and liver metastasis	UD	No	05/'01 surgery + chemo	Alive
*2*.	3 yrs	M	NB IV	Supraclavicular and BM metastases	PD	No	02/'03 surgery + chemo 07/'03 chemo, SCR + RT	Alive
*3*.	4 mnts	M	NB II	Pos. lymphnodes with unknown primary tumor	PD	No	01/'03 surgery + chemo	Alive
*4*.	5 mnts	M	NB IV S	Spine and bone/liver metastases	PD	No	01/'01 surgery + chemo	Alive
*5*.	14 mnts	F	NB II	Spine	PD	No	02/'04 surgery + chemo	Alive
*6*.	4 mnts	F	NB II	Adnex	PD	No	07/'01 surgery + chemo	Alive
*7*.	2 yrs	F	NB III	Adnex	D	No	01/'02 chemo + sugery	Alive
*8*.	3 mnts	M	NB I	Adnex	D	No	02/'02 Surgery	Alive
*9*.	2 yrs	M	NB IV	Adnex with bone/BM metastases	D^d^	No	12/'03 chemo, SCR + RT 10/'04 relapse treatment	d.o.d.
*10*.	8 mnts	F	NB III	Spine	PD	No	04/'02 surgery + chemo 12/'02 Surgery spinal relapse	Alive
*11*.	18 mnts	F	NB III	Adnex	UD^d^	20×	09/'98 MIBG, chemo + surgery 04/'99 chemo and SCR	d.o.d.
*12*.	2 yrs	M	NB IV	Adnex and bone metastases	PD^d^	37 ×	07/'98 surgery, chemo + SCR	d.o.d.
*13*.	6 yrs	M	NB III	Adnex	UD	27 ×	01/'00 surgery, chemo + SCR 11/'03 surgery, chemo + RT	d.o.d.
*14*.	19 mnts	M	NB IV	Adnex and spine metastasis	UD	49 ×	03/'98 MIBG, chemo + surgery	d.o.d.
*15*.	18 mnts	M	NB IV	Adnex and multiple distal metastases	n.d.	139 ×	06/'99 chemo + RT	d.o.d.
*16*.	16 mnts	M	NB IV	Adnex and multiple distal metastases	UD	74 ×	01/'97 chemo + surgery	d.o.d.

^a^Age at diagnosis

^b^Neuroblastoma differentiation as assessed by pathologist. D = differentiated; PD = poorly differentiated; UD = undifferentiated

^c^*MYCN *genomic amplification, as determined with qPCR

^d^Patient has been treated before surgery

BM = bone marrow; d.o.d. = death of disease; MIBG = meta-iodobenzylguanidine; SCR = stem cell rescue; RT = radiotherapy.

### ΔMYCN expression in the neuroblastoma cell line IMR-32

**Figure 2:**
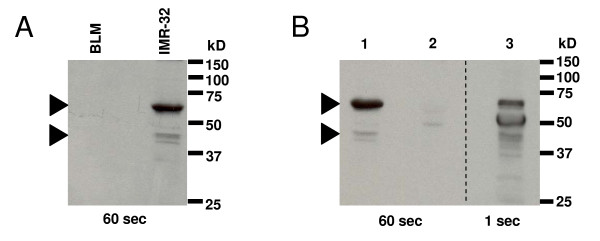
**Detection of MYCN and ΔMYCN in IMR-32 cells**. (A) Western blot, visualizing two proteins in the IMR-32 (NB cell line with *MYCN*-amplification) lysate with the C-19 antibody that recognizes the C-terminal epitope of both MYCN and ΔMYCN. In the BLM (melanoma cell line without *MYCN*-amplification) lysate, these proteins were not present. (B) Western blot of IMR-32 whole lysate (1), whole lysate minus precipitate (2) and the precipitate (3) using the C-19 antibody. Arrows indicate the positions of MYCN (65 kDa) and ΔMYCN (45 kDa). The additional band in lane 3 is caused by deposition of Ig-heavy chains (50 kDa). Exposure times are indicated below the blots.

Two splice variants have been described for the proto-oncogene *MYCN*, the classical transcript that consists of three exons and a shortened *ΔMYCN *transcript that lacks exon 2. *ΔMYCN *is expressed in several fetal tissues, but its expression has not been reported in NBs. We used primers spanning exon 2 (Figure 1A and Table 1) to visualize by reverse transcriptase PCR whether or not both transcripts are present in MYCN-amplified IMR-32 neuroblastoma cells. Two fragments were identified that corresponded with the expected product lengths of *MYCN *and *ΔMYCN *of respectively 1007 and 100 bp (Figure 1D). Sequence analyses on the excised products confirmed that these fragments were *MYCN *and *ΔMYCN*. Absence of additional fragments in the IMR-32 NB cell line suggests that there are no other major MYCN splice variants. To determine whether the ΔMYCN protein is expressed in IMR-32 NB cells, MYCN proteins were visualized with the C-19 antibody that recognizes the c-terminal epitope of both MYCN and ΔMYCN proteins. In the lysate of IMR-32 cells, two protein bands were recognized at approximately 65 and 45 kD, which are the predicted molecular weights of MYCN and ΔMYCN respectively [18] (Figure 2A). For comparison, in a lysate of the melanoma cell line (BLM), which does not carry *MYCN*-amplification, no reactivity could be observed. We conclude from these experiments that the fetal MYCN isoform ΔMYCN is co-expressed with MYCN in IMR-32 cells.

### Quantitative analyses of MYCN, ΔMYCN and MYCNOS expression levels in neuroblastomas

**Figure 3:**
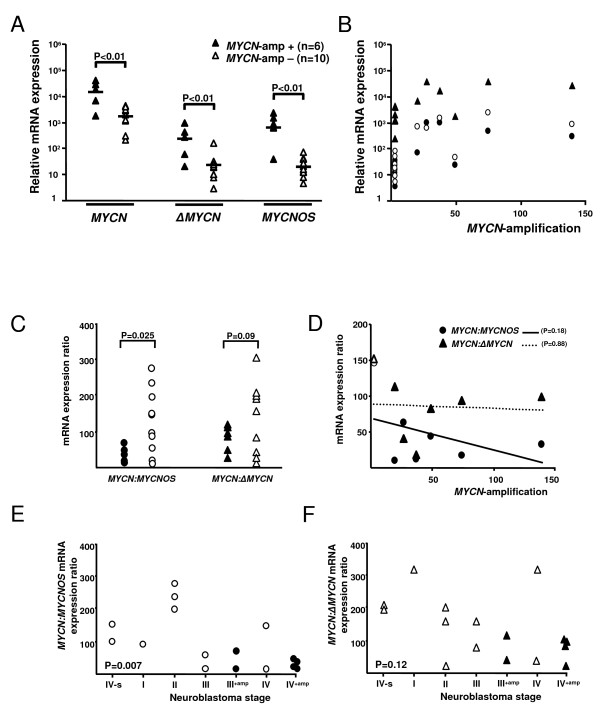
***MYCN*, *ΔMYCN*, and *MYCNOS *mRNA expression levels in NBs**. (A) Expression levels of *MYCN, ΔMYCN *and *MYCNOS *in *MYCN*-amplified (closed-triangles) compared to non-amplified (open triangles) tumours. (B) Relative expression-levels of *MYCN *(closed triangles), *ΔMYCN *(closed circles) and *MYCNOS *(open circles) correlated to *MYCN-*amplification in NBs. (C) Difference of the *MYCN:MYCNOS*-ratio between NBs with *MYCN*-amplification (closed circles) and NBs without *MYCN*-amplification (open circles). (D) Correlation of the number of *MYCN *copies with *MYCN:MYCNOS *mRNA ratios (closed circles and non-interrupted line) and with *MYCN:MYCN *mRNA ratios (open triangles and dotted line). (E) Correlation between NB stage and *MYCN:MYCNOS*-ratio. (F) Correlation between NB stage and *MYCN: ΔMYCN*-ratio. NB IV-s is a special type of NB characterized by metastatic disease with spontaneous regression and good survival [31].

mRNA expression levels of *MYCN*, *ΔMYCN *and *MYCNOS *were measured in 16 human neuroblastoma samples (Table 2) by QPCR relative to three reference genes: *GUSB, TFRC *and *RNFIII *[21]. Both *MYCN *and *MYCNOS *were found to be expressed in all 16 NBs. In addition, *ΔMYCN *expression was detected in all NB samples, except for patient 9, who did not carry an amplification of the *MYCN *region. For *MYCN*, it has been demonstrated that the relative expression-levels are significantly higher in NBs with *MYCN*-amplification as compared to non-amplified tumours [26]. Here, we show that besides *MYCN*, the relative mRNA expression levels of *ΔMYCN *and *MYCNOS *are also significantly increased in NBs with *MYCN*-amplification (p < 0.01; Figure 3A).

Correlation of NB stage with *MYCN:ΔMYCN*-ratio showed that the *MYCN:ΔMYCN*-ratio remains constant and does not change with either *MYCN*-amplification (Figure 3C; two-tailed *p*-value = 0.09, calculated with the Students' T-test) or NB stage (Figure 3F; two-tailed *p*-value = 0.12, calculated with the Spearman rank correlation). However, there is a significant correlation between the *MYCN:MYCNOS*-ratio and both *MYCN*-amplification (Figure 3C; two-tailed *p*-value = 0.025) and NB-stage (Figure 3E; two-tailed *p*-value = 0.007). The *MYCNOS:ΔMYCN*-ratio did not significantly change with either *MYCN*-amplification or NB-stage (two-tailed *p*-values = 0.58 and 0.24, respectively; data not shown). These data show that in more advanced NB tumours, mRNA expression of *MYCNO*S increases relative to *MYCN*.

### ΔMYCN and MYCNOS expression relative to level of MYCN-amplification

To more exactly determine *MYCN *copy number in the tumours that were studied, we performed a genomic quantitative PCR (gQPCR) using genomic primers recognizing five different locations within the *MYCN*-gene (Figure 1A; Table 1). Amplification of these DNA fragments was calculated relative to three reference genes elsewhere on the genome, *CFTR*, *TBX22*, and *SLC16A2*. Among these three reference genes, there were no copy number differences noted in any of the NB samples. All 10 samples with a normal *MYCN*-copy number based on Southern blotting and/or FISH, carried two to four *MYCN *copies as determined by gQPCR. The presence of a *MYCN *duplication in NB cells that lack an overt amplification of *MYCN *is more often found, although the implications for the progression of the NB are still unclear [5,27]. All 6 samples with multiple copies of *MYCN*, as determined by Southern blotting and/or FISH, had in between 20 and 139 *MYCN *gene amplifications (Table 2), which is within the normal range of gene copy numbers observed in NBs with *MYCN*-amplification [28].

We observed that *MYCN *mRNA-expression does not linearly correlate with *MYCN-*amplification, consistent with earlier reports [26]. In addition, also *ΔMYCN *and *MYCNOS *do not correlate linearly with the number of *MYCN *gene copies (Figure 3B). As shown in figure 3D, the relative mRNA expression ratio of *MYCN:MYCNOS *decreases with an increasing number of *MYCN *gene copies although this is not significant (non-interrupted line, slope = -0.4; two-tailed *p*-value = 0.18, calculated with the Pearson correlation test). The *MYCN:ΔMYCN *ratio does not change with higher *MYCN *copy numbers (Figure 3D; dotted line, slope = -0.1; two-tailed *p*-value = 0.88).

### Overexpression of *MYCNOS *in the NB cell line IMR-32

**Figure 4:**
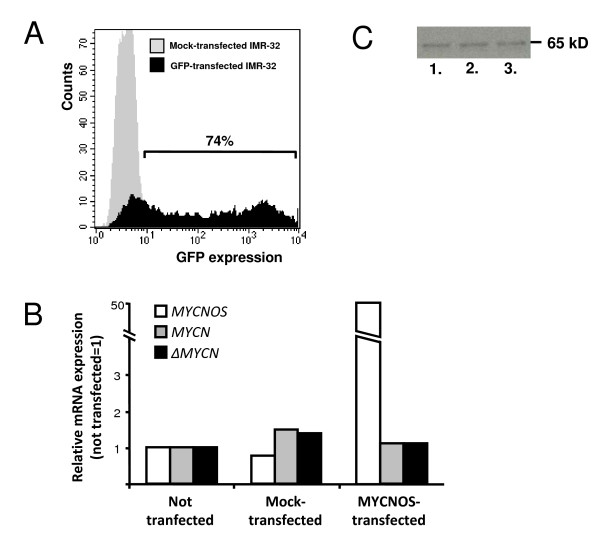
**Overexpression of *MYCNOS *in the NB cell line IMR-32**. (A) Transfection-efficiency was measured by GFP expression as analysed by flow cytometry, 74% of the NB cells expressed GFP 72 hours after transfection. (B) In the *MYCNOS *transfected IMR-32 cells, *MYCNOS *was 50× upregulated compared to not transfected and mock-transfected IMR-32 cells. Endogenous *MYCN *expression was not significantly affected. (C) Western blot showing that MYCN protein expression in *MYCNOS *transfected IMR-32 cells was unaffected. Lane 1 is loaded with lysate from untransfected cells, lane 2 with lysate from cells transfected with the empty vector, and lane 3 with lysate from *MYCNOS *transfected cells.

The pre-mRNA of *MYCNOS*, which represents the *MYCN *antisense transcript, shows overlap with the first exon of *MYCN*. Therefore *MYCNOS *may potentially modulate *MYCN *mRNA expression levels at the mRNA level via RNA-interference or RNA-editing, or direct *MYCN *splicing by RNA masking [11]. To test this premise, we transfected IMR-32 NB cells with C1-*GFP *and either the pcDNA3-vector containing *MYCNOS *or an empty vector. IMR-32 cells have relatively high endogenous expression levels of *MYCN*, *MYCNOS *and *ΔMYCN*, which enables quantification of all three mRNA levels. Flow cytometric analyses showed that there was a transfection efficiency of 74% after 72 hours (Figure 4A). Although there was a 50-fold increase of *MYCNOS *gene expression in the *MYCNOS*-transfected cell line relative to the empty vector control cell line (Figure 4B), expression of endogenous *MYCN *and *ΔMYCN *was not affected either at the mRNA level or at the protein level (Figure 4B, C). We conclude that although increased expression of MYCNOS relative to MYCN is correlated with an advanced disease state, RNA-interference or RNA-editing are not the mechanisms by which *MYCNOS *downregulates *MYCN *expression. In addition, the unchanged *MYCN:ΔMYCN *ratio in cells with *MYCNOS *overexpression shows that *MYCNOS *does not affect splicing by RNA-masking.

## Discussion

In this report, we have analysed the expression levels of *MYCN*, *ΔMYCN *and *MYCNOS *in NBs. We find that these three mRNA transcripts are expressed in NBs of all stages, but more highly in NBs with *MYCN*-amplification. The *MYCN:MYCNOS *expression level ratio is significantly decreased in high grade NBs, whereas the *MYCN:ΔMYCN *remains constant in NBs of all stages, which indicates that *MYCN *and *ΔMYCN *are co-regulated. These results suggest that *MYCNOS *might be involved in the regulation of *MYCN *expression levels as has been shown for numerous other antisense transcripts regulating expression of their sense counterparts [11,12]. However, it is important to note that the number of NB samples we investigated is relatively small. Future studies in larger cohorts of patients are needed to further establish a role for *MYCNOS *in the regulation of *MYCN *expression in patients with low-, intermediate- and high-risk NB.

Natural antisense RNA can inhibit gene expression at the DNA level by transcriptional interference or at the mRNA level by RNA-interference or RNA-editing, or regulate splicing by RNA-masking [11,12]. In RNA-masking, *MYCN-MYCNOS *duplex formation modulates RNA processing by preserving a *MYCN *population that retains intron 1, hence resulting in decreased *ΔMYCN *expression. Krystal *et al*. [29] showed that RNA-masking can occur, but they found that only approximately 5% of *MYCN *RNA interacts with *MYCNOS *RNA. Our results show that it is unlikely that *MYCNOS *expression has an effect on splicing, since 50-fold overexpression of *MYCNOS *in IMR-32 cells did not change the *MYCN*/*ΔMYCN *expression ratio at the mRNA level. Therefore, inhibition of (*Δ*)*MYCN *expression seems to be the most likely role for *MYCNOS*. There are three mechanisms by which this may be accomplished: transcriptional interference, RNA interference and RNA-editing. Since our results show that overexpression of *MYCNOS *pre-mRNA in NB cell line IMR-32 does not suppress MYCN expression, RNA-interference and RNA-editing do not seem to be the primary inhibitory mechanisms, leaving the possibility that regulation occurs at the DNA level by steric hindrance of the voluminous RNA-polymerase complexes on opposite DNA strands.

It is not clear how increased expression of *MYCNOS *contributes to the development of NB. Although the increase of *MYCNOS *expression levels is higher than that of *MYCN *in NB with amplification, this difference does not appear to influence the prognosis of patients. In patients with NB but without *MYCN-*amplification, it would be interesting to investigate whether the *MYCN:MYCNOS *ratio is a good prognostic marker. Differences in *MYCNOS *expression levels might explain some of the controversies about *MYCN *expression and prognosis of these patients [1-3].

Besides *MYCN *and *MYCNOS*, *ΔMYCN*, which was previously identified as a fetal transcript [18], is also expressed in NBs. No other *MYCN *isoforms were detected. This suggests that the alternative splice variant that previously has been described by Stanton et al. [30] and consists of an alternatively spliced exon 1, has little relevance in the progression of NBs. In one tumour, the *ΔMYCN *transcript could not be identified, but this is probably because *ΔMYCN *is low expressed in general and in this tumour *MYCN *was not amplified. The ΔMYCN protein contains a nuclear localization signal, a basic helix-loop-helix, and a leucine-zipper domain, which may serve to dimerize with MYCN or bind to its DNA binding site. ΔMYCN lacks the transactivation domain including the highly conserved Myc 1 and 2 boxes, from which it was speculated that it competes with MYCN and therefore inhibits the active MYCN protein [18]. However, in all neuroblastoma samples analyzed, the ratio between MYCN and ΔMYCN expression remains constant and does not correlate with *MYCN*-amplification or disease stage, indicating that ΔMYCN induced inhibition of MYCN at the protein level is not of relevance in NB.

## Conclusion

In conclusion, our results suggest that the expression of the antisense gene *MYCNOS *might be relevant to the progression of NB, potentially by directly inhibiting *MYCN *transcription by transcriptional interference at the DNA level. Analysis of *MYCN:MYCNOS *expression ratios in patients with NB without *MYCN-*amplification and clinical follow-up are necessary to establish the relevance of *MYCNOS *expression to the prognosis of these patients.

## Competing interests

The authors declare that they have no competing interests.

## Authors' contributions

JFMJ and APMB designed, performed and analyzed the research and drafted the manuscript. HB and PH conceived of the study. FNL supervised the transfection experiments and helped to draft the manuscript. CAHK performed all pathological characterizations. HB, PMH, GJA and IJMV conducted the study as the principal investigators and contributed to the preparation of the manuscript. All authors read and approved the final manuscript.

## Pre-publication history

The pre-publication history for this paper can be accessed here:

http://www.biomedcentral.com/1471-2407/9/239/prepub

## Supplementary Material

Additional file 1 **Supplemental figure**. Ponceau S stainings of the immunoblot shown in (A) figure 2a and (B) 4c Click here for file
