# Supplementary figures and images for: Regulation of MYCN expression in human neuroblastoma cells
Source: BMC Cancer. 2009 Jul 18;9:239. doi: 10.1186/1471-2407-9-239 (PMC2720985; doi:10.1186/1471-2407-9-239)

## Slide 1
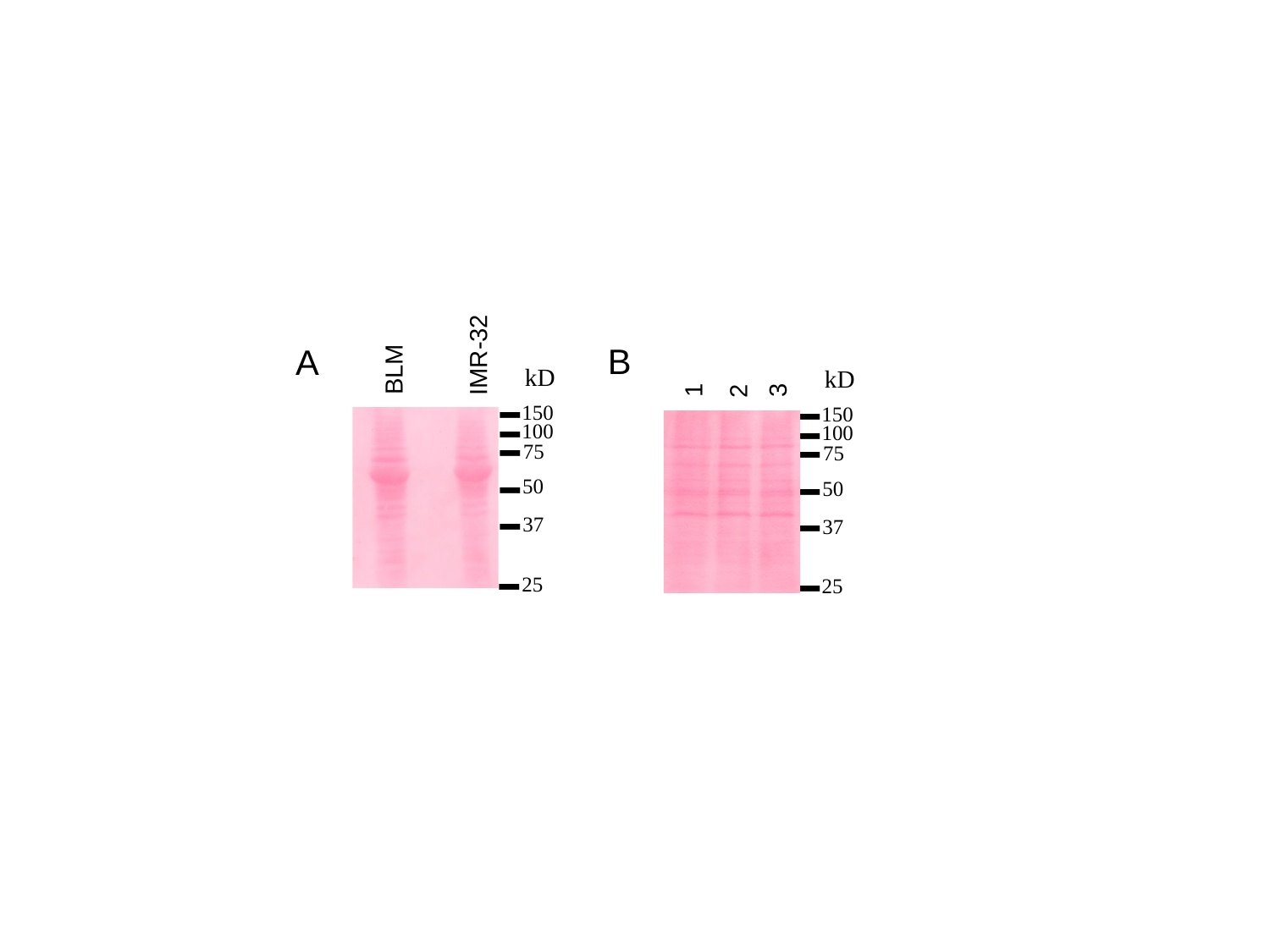

IMR-32
BLM
-
kD
-
-
150
100
-
75
-
50
37
-
25
3
2
1
-
kD
-
-
150
100
-
75
-
50
37
-
25
B
A

Supplement: Additional file 1 — Supplemental figure. Ponceau S stainings of the immunoblot shown in (A) figure 2a and (B) 4c [file 1471-2407-9-239-S1.ppt]
